# The Study of Diseases of Children

**Published:** 1882-12

**Authors:** Wm. B. Atkinson


### ARTICLE II.

### *The Study of Diseases of Children.*

BY WM. B. ATKINSON, M. D.

I have selected as the theme of my lecture this subject, because I have long been impressed with its importance, and feel that it cannot be too frequently or too forcibly placed before the profession.

Children, the fountain, the origin of society, those who are to build up the body politic, must be healthy or we can not hope to have them grow up to a healthy adult age. It is appalling to every American who studies the subject to find that the native born is rapidly becoming outnumbered by the alien. One great cause of this state of affairs is too well known to require mention here. But another cause, perhaps equally potent, is the neglect of the proper study of childhood and its diseases, and hence the great mortality in this class. I say the great mortality, for though we must admit that great strides have been made within the last few decades in the way of lessening this mortality, yet it remains painfully great. Observe the list of deaths in any of our great centres of population, and we are at once struck by the immense numbers of children. Now, when we reflect upon the former belief, held most universally, that it was virtually useless to hope to lessen this proportion, do we not have room to hope for a continuance of the improvement? Perhaps much of this mortality is due to neglect of proper sanitation on the part of the parents; and while much has been done to instruct them in this particular, there remains much to be done, not only in the way of instruction, but even by compulsory measures, that these little ones may be saved from the terrible holocaust. This work is eminently that of the physician—the caretaker—whose duty it becomes to prevent disease rather than to cure it. We should constantly instruct parents as to the value to their little ones of pure air, sunlight, cleanliness, proper food and exercise.

Perhaps, however, we may look a little closer home, and learn why these matters are so frequently neglected. For some reason the study of children and their ailments has generally been but little cultivated by the profession. We find this in our medical schools. Until very recently, while the chair of obstetrics included the study of women and children, the latter subject was so completely ignored that there was scarcely an illusion made to it. The chair of practice indeed treated of a few of the diseases incident to childhood, but their study as a special branch was never regarded; and though in a number of schools chairs have been made solely devoted to the subjeet, yet the attendance of the student upon these lectures is by no means obligatory, and he is left to his own choice in the matter. Fortunately, many young men are impressed with the importance of the subject, and the class usually give full attention to this part of the course.

Engaged as I have been for so many years in this specialty, I have become forcibly impressed with some causes for the neglect of this study.

By many it is believed that it is extremely difficult if not impossible in many instances to ascertain the pathological condition of a child. Particularly is this maintained in the cases of very young children who are unable to make known their feelings. Now, we insist on the contrary, that by a careful investigation we may learn with greater readiness the true state of health, not by inquiry of the little patient, but by a rigid comparison of its objective symptoms with those which should obtain in a healthy child of the same age. We are less likely to be misled than in the cases of adults, who, involuntary it may be, exaggerate their feelings, or reply to our questions in such a way as to prove the folly of making our inquiries so that an affirmative answer seems to be expected. In fact, we frequently find this carried to such an extent as to make the whole examination a farce. Often a person will, by these replies, have diarrhœa and constipation, be sleepless and too drowsy, have pains everywhere, and yet by her actions prove herself able to move about without the slightest inconvenience. A literal report of the questions and replies at some of our clinics would be regarded as a caricature, and it generally requires a most thorough sifting to get at the exact truth, and this, too, when there exists no reason for any deception.

On the contrary, the baby, when properly interrogated, replies in such a way that no deception is possible.

Perhaps one great cause of the difficulty in the diagnosis in children, especially the very young is the utter want of knowledge as to what constitutes a healthy child. In my contact with students, and even with older members of the profession, I have often been surprised at the ignorance shown as to many of the conditions of child-life.

Thus it becomes necessary for us to know what would be the normal state of a new-born child—as to its average size, weight, general appearance, pulse, respiration.

In this connection, fancy the error of a father who was panic-stricken to find that his month-old baby had aggravated palpitation of the heart, and his relief on being informed by the physician whom he had summoned that the normal pulse of a child at that age varied greatly from that of his own pulse.

The physician should not pronounce an infant to be suffering from diarrhœa because in its first months it has frequent evacuations.

He should know that it does not secrete saliva, and that every act of regurgitation from the overloaded stomach is not vomiting, so to speak, but is merely a wise provision of nature to prevent serious trouble in the alimentary tract or by reflex irritation elsewhere.

He should be informed by the normal frequency of its taking the breast, and the quantity taken at each time, that he may instruct the mother or nurse in those unfortunate cases where artificial feeding becomes imperative.

In short, let him learn all that can be learned about a healthy infant from the moment of birth, and trace its progress day by day, month by month, year by year, until it ceases to belong to the class of children, and takes its position as a mature individual.

With a thorough knowledge of all these points, he is now prepared to draw the line between health and disease, and to decide with more prospect of success as to the indications demanding medication or its omission.

Nor is this all, he must learn that, particularly in children, symptoms which often appear very grave are evanescent, and, therefore, must be guarded both as to his prognosis and as to the quantiiy of a remedy which he may order. You can understand this latter allusion when you encounter a case in which a physician, no longer a neophyte, has written a prescription for a four ounce mixture and gravely informed the parents that the child was in a very critical condition, and who returned the next day to find the patient playing on the floor, and the bottle scarcely touched as to its contents.

The reverse obtains in the following instance. Two, children, a boy and girl, were attacked with diphtheria. After some days a consultation was called, solely because the boy failed to build up. The latter could scarcely convince either the physician or the parents that the child was dying; and it did die, spite of stimulation and every effort that could be made, within a few hours.

Nor is this all, the girl, a year or two younger, some two or three days later, was placed at the table to eat its breakfast, with the idea that this would tempt it to eat more heartily. In attempting to swallow it chocked, and the physician coming in shortly, was informed that "some of the food had gone the wrong way," and he failed to recognize the presence of analysis of the organs of deglutition until he was shown this condition by another, Here, again, death soon closed the scene.

Each case, each child, shouid be studied by itself. Its antecedents should be learned, every point in its history noted—its peculiarities, its surroundings, its relations.

No haste should be exhibited in arriving at a diagnosis, nor should we refuse to listen to all the information vouchsafed by the mother or nurse. Frequently the clue is thus obtained and the knot untangled. Thus the annoyance might be spared which on one occasion occurred when the doctor pronounced it a mild case of "rubeola," while the old nurse said she never heard it called anything but "gum," and the result proved she was correct.

Inspect the child thoroughly, both awake and asleep, and particularly where the symptoms are obscure should it be examined undressed. The importance of this last will be seen by allusion to a case. A mother brought to me a child aged between six and seven months. It appeared rosy, lively, healthy—natural in every way except that it would occasionally cry out in great pain. She informed me that two or three doctors had given her things to relieve it, but without effect. Failing to find anything to account for this sudden outcry, I requested her to remove its clothing. This was done with great gentleness, but I observed that the child screamed as it was moved. Almost as soon as its legs were exposed I recognized the trouble. There was a partial fracture of the thigh about the centre, and when the proper dressing was applied so as to prevent the motion of the broken bone the outcries ceased, and the baby was soon as well as ever. No doubt this was due to muscular contraction, as no history of any injury could be obtained.

Undressing the child not only exposes every part to view, but also gives an opportunity of seeing whether any part of the dress is too tight or not properly adjusted. Hence it behooves the physician to watch the process, though he may do this in a way not to attrct the attention of the nurse. He may thus detect errors which she would prefer to conceal.

Learn as to its diet. If hand-fed, inquire as to the amount given, the way it is administered, the proportion of water or other diluent. The latter is a most important item, as we constantly find our little patients starving slowly when they are supposed to be taking an abundance. A diet composed of one part milk and two parts water will not support a child. Under such a regimen it is always hungry, while the mother fondly, though ignorantly, believes that she is giving it a full supply.

In this connection we may allude to the need of an examination of the mother's milk when the child is failing in health. Iuquire as to her health, whether she is pregnant or menstruating, for it is well known that both of these conditions tend to reduce the nutritive or healthful quality of the milk ; and though she may appear to have the usual amount for her infant, it is so deteriorated that it fails to properly nourish the child, and frequently acts as an irritant to the stomach and bowels. Again, the habits of the mother are often of importance in giving aid in the diagnosis and treatment. Her occupation, her surroundings should be understood.

A case will illustrate this point. A child at the breast was suddenly attacked with apparent coma. By the time the physician arrived this was passing off, and no symptoms were present which could account for the attack. The mystery was readily explained when it was learned that the mother had been washing all the morning, and was so anxious to conclude her task that she did not either stop either to give herself or her child any nourishment. The moment she had finished, she had sat down, heated, exhausted, and placed the child to the breast with the result mentioned.

Anger, fright, any excitement, is always likely, and rarely fails, to produce evil effects upon the milk of a nursing woman, and she should be strictly charged as to suckling the child immediately after such occurrence. Perhaps many of us can recall instances where children have been seized with convulsions, etc., under such circumstances, and I doubt not that many of the ephemeral attacks to which children are subject are the result of such a course. Fortunately, nature in the case of children has great recuperative powers, and if left alone will often restore the child to its normal condition.

This point is one upon which I cannot lay too much emphasis—the power of nature to restore health. Perhaps this alone is the true secret why many, otherwise ignorant, and a certain set of practitioners are so successful in the treatment of disease. They say let the case alone, or leave nature to do the work. Medicine is given which is really nothing. Rest and the most rigid diet are enjoined, and thus nature is not interfered with, and proceeds in her own way to restore the health. We should be content in every instance to act with our medicine solely to meet a positive indication. We are only the assistants of nature; we have not the "healing power in our hand," and it becomes our duty to act by removing causes where they can be reached, to relieve pain, and particularly to see that those who are administering to the sick do not by their officious kindness do too much, and thus interfere with the natural return to health. Perhaps one most important point for the young physician to learn at the very outset is that drugs are not all-powerful. That time, rest, diet, and numberless little things are truly the means by which we aid in the fight against disease.

Time is important, and we may illustrate this by the cases which we constantly encounter, where a child has a moderate diarrhœa, which, by care and appropriate regimen, would in a reasonable time disappear. But in our haste to cure we exhibit astringents, stimulants, narcotics, and a host of articles, often making the case much more serious than in the beginning, and nearly always retarding the progress to health.

One of the earliest duties in caring for a case of sickness in a child is to observe the indications which present and act accordingly. Thus, in a diarrhœa, he should know for himself the exact appearance of the stools, their color, consistence, quality; the presence of blood, mucus, foreign matters, membranous shreds; frequency, the presence of pain, before, during, or after evacuation; whether there is also vomiting, abdominal tenderness, acidity of the evacuation, unusual fetor; each of these points will give him an indication to meet in the effort to correct the abnormal condition, and generally the next twenty-four hours will show an improvement.

In very many cases it is an excellent plan not to continue the medicine too long. Place the child on the road to health, and see if with a little supervision it cannot continue to improve. But do not too soon, while discontinuing the drugs, abandon the case as to diet, rest, etc. Some people imagine the moment they cease to use medicine they are well, and can at once return to their former habits. Hence the frequent relapses in disease, and the necessity for enjoining most earnestly that no departure be made from the strict plan laid out until the physician allows it.

Here we may allude to the great value of change of air and change of scene. Not only is this important in cases where the child is located in a blind alley, cut off from the sunlight and fresh air, surrounded by filth and decay, but it has been found of almost equal service in cases where it might be supposed that there was all that wealth could procure. There are so many subtle influences working quietly, yet effectually, to undermine the health, that we cannot always understand the origin of disease or the causes of its continuance. Hence the value of a change if only to another locality. The improvement in many instances is immediate. This may be due greatly to the better air, cooler temperature, etc., and surely we must see the same effect upon children that we do it in adults. Frequently the system is roused as it were from an apathetic state, and stimulated to new efforts at recuperation. In several cases, after a child has been for several days lying without any apparent improvement, convalescence has followed a change from one room to another. I shall not attempt to explain this, but the fact is there, and is worthy of attention when the circumstances will permit of it.

This, too, holds good in the beginning of illness The old lesson so frequently given us to oppose the beginnings is equally true in regard to the health. The apparently trifling symptoms of to-day may develop into the full fledged attack of to-morrow. Hence it becomes the duty of the physician to impress upon the parents the necessity of attention to every untoward symptom. I would not have them magnify such matters, but we all can readily recall instances where serious trouble has resulted from such carelessness. The child is out of sorts, does not eat, is irritable, refuses to play, or quickly abandons one thing for another, these actions should arouse our suspicion, and an effort should be made to ascertain the cause. A case has recently occurred to me with just such symptoms as the above. They were disregarded as the result of "crossness." On the third day convulsions set in, which continued without cessation until death closed the scene.

Symptoms such as these, while squinting, frowning in the sleep, rolling the head, vomiting and general constipation, can never be neglected with impunity. In addition, we have turning in of the thumbs which is almost invariably associated with brain trouble.

I cannot leave this point without allusion to those cases where the child halts in its walk, or returns to crawling after it has walked. Such symptoms should always arouse suspicion, lest they may be the early warnings of hip-disease or spinal affection.

We constantly find it true that the parents are too apt, after many scares, to go to the other extreme, and neglect calling the physician until serious injury has occurred.

Perhaps it would be well at the outset of our study to understand why some persons are more successful with children than others. That is, that they can with more ease approach a child and ascertain the condition. It should be remembered at the outset that children are great observers. Instinctively they know who approaches them gently, kindly. The very young child is easily startled, and it behooves the physician to act with the utmost circumspection when he makes his first visit. Let him at once seize the child and endeavor to feel its pulse, look at its tongue, or examine it in any part, and he immediately arouses a fear, a suspicion, it may be a feeling of antagonism in the child, which will take a very long time to subdue. On the contrary, he is wisest who acts as though the child was not to be the subject of inquiry. A conversation with the parent or nurse, very guarded as to the child, who should all the time object of examination in its every movement, will generally place it more at its ease. The strange man is to it an object of curiosity; it wants to know whether he is to be feared or opproached. Like the antelope on the plain, the child is largely endowed with curiosity, and as soon as it finds there is nothing to dread—no repulsion—it is attracted, and desires to learn more of him. In this way the sensible physician speedily places himself on a pleasant footing with his little patient, and often before he knows how it has occurred he has the infant in his arms, feels its pulse, hears the action of the heart and lungs, knows the temperature, the condition of the skin, and has made a good many steps in his diagnosis.

To feel the teeth to see the tongue, to ascertain the state of the throat, are matters as readily accomplished. The finger dipped in a little sugar, if need be, can without difficulty be passed into the mouth—the small finger preferably; the condition of the gums, the presence of teeth, are noted, and then, being passed a little further back, the child gags, and the throat and mouth are quickly inspected and thus a a seemingly difficult task is performed. Who can fail to be astonished at the trouble experienced by the man who approaches a child of any age with a spatula or spoon? Couple this with a demand to "open your mouth," "put out your tongue," and the child is seized with a fear of something that is to follow which arouses its little powers of resistance. And if, finally, the physician obtains his purpose—he often fails—he has done so much harm that he usually adds to the original sickness. Should the manœuvre as above be successful he must be prepared to learn in a quick glance all that he wants about the mouth and throat—whether there be aphthæ, inflammed gums, sore throat, diphtheritic ulcers. Knowing the normal color of these parts, he can tell whether they are blanched or conjested. On the subject of sore throat, a simple action always gives us a clew. Let the child swallow a sup or more of cold water. If it swallows without hesitation drinking readily, the throat is in proper condition.

On the subject of a proper approach to a patient, I have often wondered how some men can obtain practice. It is imperative that in every way we should commend ourselves to these little people, and is it not equally so with regard to patients of older growth? Can the physician who comes to the sick-bed—say of a delicate woman—with clothing fumigated with the fumes of tobacco, perhaps even laying the cigar still smoking on the table while he talks; or, what is worse, examining the tongue or throat, while every breath is loaded with whiskey, be otherwise than offensive? It is a terrible misfortune when one has a bad breath the result of disease, yet how many of us work incessantly to acquire one!

I trust you will pardon me for this digression, but I have so thoroughly been imbued with the relief that the physician should be a scholar and a gentleman, that I can never view him otherwise without a feeling that he has aided to degrade the noblest of callings.

To return to my subject: the examination may, except in rare cases, be made a sort of play with the child. He is tickled and the laugh hurts him; a clue is obtained. He speaks or cries; the tones tell us much as to the throat and lungs. His decubitus shows whether pain prevents the proper position. In short, with a proper knowledge of the health, and a careful inspection of every movement, one often can say to the anxious mother much to encourage her, or to assure her that she has not failed in securing the services of one skilled in his profession.

Having learned as far as we may the diseased condition of our child, our next step is to remedy it, and restore it to its normal state. In attempting this let us "make haste slowly," lest in our hurry, in our eagerness to apply our remedies, we not only interfere with nature's efforts but actually add to the present trouble. Fortunately, the days of bleeding and actively physicking children have passed never to return. We no longer aid disease by reducing the child's strength to resist it.

In medicating children remember what I said at an early point. Ascertain the indications, and endeavor to meet them. Is the stomach loaded with indigestible or poisonous matter? Empty it with an emetic. The one most handy and efficacious will be of mustard or salt and warm water, followed by plenty of warm water, to thoroughly wash out the offending substance. If the case has been so delayed that much of the mass has passed into the bowels, give a brisk purgative, and a few injections into the lower bowel of warm water, with soap, and which may be added castor oil.

If there is reflex irritation, with convulsive tendency, cinapisms to the spine and elsewhere should at once be employed.

If fever is present, tepid sponging almost invariably reduces the temperature, and cool acid drinks greatly aid in giving speedy and positive relieve.

Employ always the mildest remedies at first, and aid their action by quiet, rest, and diet. But do not fear when the emergency demands to use those articles which experience has shown to have power to meet such an emergency. Invariably exhibit such medicines in the minimum dose, increasing or repeating until the desired effect is obtained. Thus, as an illustration, we may take a case of convulsions, which to every one concerned is usually a source of great anxiety or excitement.

If called during the convulsion I would employ the hot bath; cold to the head, particularly in the form of ice in the bladder, thus getting all the cooling without so much of the wetting effect; broad sinapisms the whole length of the spine; and, unless the spasm quickly ceased, anæsthetics by inhalation. An interval having been secured, act according to indications. Should there be reason to regard the convulsion as the result of loaded stomach or bowels, relieve both as before mentioned, and quiet the tendency to a return by bromide or chloral, or both. I may here mention that the latter may be very usefully thrown into the bowel, and thus avoid its unpleasant effect when administered through the mouth. Or, if there is reason to regard the spasm as a prodrome of one of the eruptive fevers, its recurrence need hardly be apprehended, and the case should be watched for other symptoms to be treated as they arise.

Perhaps in no case do we require more care in making the diagnosis. Such attacks are often the result of injuries to the brain, exposure to the sun, the result of heart-stroke, or a symptom of meningeal or cerebral congestion or inflammation. Fortunately, rest, quiet, and the treatment already mentioned, are the best that we can do until positive indications arise which demand other remedies.

Upon another point it becomes important that we should dwell for a little—the necessity of support by appropriate diet, or even by tonics, during the continuance of disease. A widely diffused belief, but one very erroneous, is that milk should be withheld from a child during fever. We have but to point to the constant suckling of a child and its necessity to prove this error. See to it that amid the care to administer medicine, that food is not neglected; that it be given in proper form and quantity, and at the proper interval. In many of the affections of children—say measles or variola—much evil is caused by the great reduction of the strength, both by reduced diet, and by reducing medicines. Where children have a predisposition to phthisis, etc., it wants but this to arouse the latent disease, and the child too often recovers from the one only to be attacked and speedily carried off by something more dangerous.

Medicines for children should always be exhibited in a palatable form. Pills, especially, should be avoided. Disguise the remedies by the employment of pleasant syrups, and make the quantity to be taken as small as may be. Frequently a pleasant remedy like the liquor potassæ citratis, with syrup of lemon or the like, will cure a moderate fever, or act as a placebo until there is more urgent need.

Perhaps my lecture would be incomplete did I not allude to the necessity, too frequently occurring, of meeting an annoying indication. I mean the determination on the part of so many who have the care of children that they shall take medicine because they are supposed to require it. The child picks its nose, and has other valuable symptoms of the presence of worms, hence it must have some worm medicine. The doctor is appealed to, and if he, very injudiciously, pooh poohs the matter, the drug store is sought, and worm lozenges, teas, or other injurious trash, are bought of the complaisant apothecary, and the child is often rendered really ill by this administration. Where we have to do with people of sound judgment it is better to explain to them these matters at some length, and we can generally convince them.

Unfortunately, however, every neighbor must be consulted, and the doctor is either voted an ignoramus or an old fogy, and the result is generally a very sick child from such officiousness.

Often it has been better to appear to yield, and give a placebo as above, and thus save the child from a worse fate.

In assuming the charge of a child, it should be an early duty of the physician to obtain information as to what has been the habit of the mother: if she has accustomed it to use of soothing syrups, etc. Many cases, otherwise obscure, are made plain when we have obtained an honest history from those who have them in charge. Insist upon this in every case, for very many do not scruple to employ these articles for the most selfish purposes, and yet are ashamed to have it known to the physician. You can rarely hope to get such people to abandon their use, and can only employ their knowledge to enable you to employ the appropriate treatment when disease occurs.

In these days, when the cares of maturnity are to be avoided as much as possible, and where infanticide is frequently preferred, we cannot ask that those who submit to their lot and go through the pangs of labor shall be further annoyed by sleepless nights, or prevented from enjoying their regular round of pleasure merely by a crying child. And when they are presented on every side with such palatable articles which the child itself learns to cry for, it is really inhuman to expect that they will refrain from doing what every one does, even though it should cause the stunting of a child, or aid in swelling the infatile mortality.

Amid the great demorilization of the day, ours would appear almost a hopeless task. But we cannot lay it down any more than the sanitarian who so constantly finds his best efforts frustrated by greed, avarice, ignorance, and the host of obstacles always in the way of right and truth.

And now, gentlemen, in concluding, let me hope that I have not detained you to no purpose. I trust that I have sown the seed, planted some germs, that may grow and yield a harvest of good to the cause.—*Medical Bulletin.*

---

